# The earlier you know, the smoother you act: anticipatory control in solo and dyadic juggling

**DOI:** 10.1007/s00221-026-07311-z

**Published:** 2026-05-12

**Authors:** Abir Chowdhury, Heiko Maurer, Alap Kshirsagar, Kai Ploeger, Jan Peters, Hermann Müller, Lisa Katharina Maurer

**Affiliations:** 1 Neuromotor Behavior Laboratory, Department of Psychology and Sport Science, Justus Liebig University, Giessen, Germany; 2 Center for Mind, Brain and Behavior, Universities of Giessen, Marburg and Darmstadt, Giessen, Germany; 3 Intelligent Autonomous Systems Group, Department of Computer Science, Technical University of Darmstadt, Darmstadt, Germany; 4 German Research Center for AI (DFKI), Darmstadt, Germany; 5 Hessian Center for Artificial Intelligence (Hessian.AI), Darmstadt, Germany; 6 Institute for Sports Science, University of Kassel, Kassel, Germany

**Keywords:** Interception, Minimum jerk trajectory, Toss juggling, Internal model, Trajectory planning, Predictive control

## Abstract

Intercepting a moving object with our hands requires that the moving object and the hand come into contact at some point along the object’s trajectory. Hand movement can be planned in advance so that the hand reaches the interception location along this trajectory at the correct moment in time. To enable such anticipatory behavior, the object’s trajectory may be predicted based on sensory information collected before movement planning is concluded. Early completion of planning would allow for a smooth and economical movement; however, it also requires sufficiently accurate information about the object’s movement to be available earlier. We investigate interception in the context of a complex, naturalistic motor task, toss juggling, to understand the extent to which proprioceptive and tactile information influence anticipatory movements of the catching hand. In our study, we compared solo juggling, where the efferent, tactile and proprioceptive information from the throwing hand is available during catch planning, with dyadic juggling, where no such information is available because the ball has been thrown by another person. Our results indicate that when internal and tactile information from the toss is absent, anticipatory behavior is significantly reduced. Toss jugglers seem to rely more strongly on internal information than vision, allowing earlier planning and lower target uncertainty. In the dyadic condition, when internal information is absent, trajectory planning is delayed, leading to greater target uncertainty.

**Supplementary Information:**

The online version contains supplementary material available at 10.1007/s00221-026-07311-z.

## Introduction

Interception is a fundamental component of everyday motor tasks, particularly in dynamic activities such as hitting a moving ball in sports or, in more static contexts, reaching for a glass on a table. In the context of a dynamic activity, it can be described as the process of coordinating spatial and temporal parameters to arrive at a specific location at the precise moment another object reaches that point, thereby enabling physical interaction or contact with the object. In ball-catching tasks, the brain uses sensory information about the ball’s movement to generate predictive estimates of its future trajectory, enabling anticipatory motor planning (Hayhoe and Ballard 2005; Diaz et al. 2013; de Lussanet et al. 2004; Park et al. 2023). However, a large body of research also supports the idea that successful interception can be achieved without explicit predictive computations. For instance, fielders catching fly balls and players intercepting fast table tennis shots adjust their movements prospectively by coupling their actions to continuous optical variables, such as image speed or time-to-contact (Michaels and Oudejans 1992; Bootsma and van Wieringen 1990). Similarly, changes in a ball’s path have been shown to result in immediate kinematic adaptations during catching, supporting dynamic, real-time control over pre-planned trajectories (Peper et al. 1994). Even under conditions where acceleration is hard to judge, performance improves through trial-by-trial corrections rather than from accurate internal predictions (Brenner et al. 2016). In line with this, Katsumata and Russell (2012) found that participants hitting a falling ball could successfully time their actions using continuous visual cues, even in the absence of detailed predictive information, further supporting the role of prospective, online control in interception. Together, these findings suggest that the balance between prospective and predictive control may depend on task demands, available sensory information, and individual differences (Zhao and Warren 2015; Arthur et al. 2024; Márquez et al. 2024; Sun and Jang 2025). From these we are mainly interested in how the available sensory information is used based on the expertise of the individuals.

Prospective control emphasizes the continuous coupling of action to visually specified information from moving targets. However, in juggling, the target itself is in motion, and time constraints may limit the contribution of prospective control during the final phase of the hand movement, increasing reliance on predictive planning. Therefore, interception in this context might not be fully explained by continuous online control alone. According to this view, the brain forms internal models that predict the sensory consequences of motor commands (Wolpert et al. 1995, 1998; Kawato 1999), enabling anticipatory control even before sensory feedback is available. Toss juggling is an extreme example of a task where such internal models could be essential, as it demands rhythmic catching and throwing of objects under tight spatial and temporal constraints (Beek and Turvey 1992). The dense time schedule of juggling requires the juggler to initiate the final approach toward the interception location, while simultaneously coordinating the throw of the ball already in hand. Unlike discrete object-catching, where the hand often comes to a stop after interception; juggling involves continuous motion, each catch serves as a preparation for the next throw. For instance, in a cascade pattern, this seamless coordination is crucial for maintaining a stable juggling rhythm, as the quality of each throw directly affects the ease of the following catch of the same object by the other hand. After all, the difficulty in juggling increases due to the simultaneous control of multiple objects. However, balls are not thrown to random locations; rather, the trajectory of each throw is structured and predictable. As a result, each ball need not be visually tracked or caught independently. Jugglers capitalize on this predictability by relying on the accuracy of their throws and internal predictions of where each ball will travel. This reliance on internal models over continuous visual tracking is perhaps most strikingly illustrated by the fact that up to five balls can be juggled while blindfolded (Botvinick-Greenhouse and Shinbrot 2020). The degree of accuracy required in these throws reflects the inherent instability, and thus the difficulty, of a given juggling pattern. Experienced jugglers further optimize performance by fixating near the apex of the juggling arc, rather than following each ball individually (Dessing et al. 2012, 2009). This gaze strategy, along with multisensory integration from proprioceptive information about the own movements as well as tactile and visual information about the ball’s behavior and a finely tuned internal model, enables them to maintain control and rhythm in an otherwise highly unstable and dynamic motor task. This capacity to integrate multiple actions into a continuous process distinguishes experienced jugglers from novices, who often exhibit disjointed and reactive movements. Ultimately, juggling exemplifies a highly efficient form of motor control in which multiple actions are anticipated and executed with precision to sustain uninterrupted motion.

One way to analyze the different components of coordinated juggling and their interrelations is to use the minimum jerk trajectory-based approach proposed by Slupinski et al. (2018). With this approach the planning process of catching can be computed from the shape of the hand-movement trajectory. The fluidity in the catching movement is achieved through a combination of feedforward control, which enables prediction-based adjustments before the ball arrives, and feedback control, which allows for real-time corrections when deviations occur. This characteristic is manifested in a bell-shaped velocity profile typical for goal-directed reaches. The term “minimum jerk trajectory” refers to the smoothest possible movement between two points over a given time: it minimizes the integral of squared jerk (the rate of change of acceleration) (Flash and Hogan 1985). This optimal control-based mathematical model has been widely used in the literature to characterize predictive, feedforward execution (Shadmehr and Wise 2004; Huh and Sejnowski 2015; Fligge et al. 2012; Richardson and Flash 2002). Following Slupinski et al. (2018) and Hagenfeld et al. (2022), we interpret the onset of the smooth-approximation phase as reflecting when the catcher’s prediction of the ball’s future trajectory has become sufficiently accurate that corrective adjustments cease. An earlier onset therefore indicates earlier availability and integration of predictive information, whereas a later onset reflects continued reliance on incoming sensory information before the movement settles into a smooth trajectory. By relating the timing of this smooth-approximation onset to the release time of the toss from the other hand, we can estimate when the catching hand begins to follow a smooth path toward the ball, serving as a proxy for the completion of planning for the initial ballistic approach to the catch.

Previous work has identified the onset of smooth, goal-directed movements in discrete interception tasks using self vs non-self throws (Slupinski et al. 2018; Hagenfeld et al. 2022). In the present study, we investigate movement planning in a more complex interception task, toss juggling, where the hand transitions fluidly between throw and catch phases. From the hand’s perspective, the process of catching a ball during juggling involves continuously adjusting its movement to get closer to where the ball is expected to be. At each moment, we can calculate the shortest distance between the hand’s position and the predicted path of the ball. This creates a distance profile that shows how the hand approaches the ball’s trajectory over time. The shape of this profile reflects how the hand prepares for and moves toward the catch, giving insight into how the juggler plans and controls their movements. Looking at the process this way highlights how the hand actively works to meet the ball’s path, rather than just reacting to where the ball ends up.

We compared two juggling conditions, solo 3-ball cascade juggling (condition 1) and dyadic 3-ball cascade juggling (condition 2), with the independent variable being the availability of proprioceptive and tactile information from the throwing hand. Following the approach of Slupinski et al. (2018), we expected that jugglers can predict a ball’s trajectory earlier in solo juggling compared to dyadic juggling, and that the duration of the planned phase of the movement toward interception is longer in the solo case. This serves as our dependent variable. To our knowledge, this is the first study to examine both solo and dyadic juggling and the influence of internal information on the planning process during ball catching in toss juggling. Interestingly, the dyadic scenario leads to larger flight distances and, consequently, longer flight times. This aspect has been taken into account in the analyses presented later in the paper.

## Materials and methods

### Participants

A total of 18 jugglers (12 males and 6 females) participated in the experiment. The jugglers’ ages were in the range 20–60 years (mean = 30 years, SD = 10.42 years). The jugglers were chosen based on the inclusion criteria that they are able to comfortably sustain a 3 ball cascade pattern for longer than 20 s. Prior to the experiment, all participants completed a questionnaire assessing their juggling experience and the patterns they could perform along with a personal evaluation. With the exception of three participants who reported being able to perform more advanced juggling patterns (e.g., a 5 ball cascade and a 3 ball Mill’s Mess), all participants indicated that they could comfortably juggle a 3 ball cascade pattern. All participants were either ambidextrous or right-handed. Handedness was assessed based on the participants’ preferred hand for daily activities and writing. None of the participants knew one another personally.

The participants received a monetary compensation of 8 € per hour or course credits, whichever they chose. The study was conducted following the Declaration of Helsinki 1964 (Association 2025). The protocol was approved by the Ethical Review Board of the Justus Liebig University, Giessen, and subjects gave written informed consent to participate in the study.

### Task and setup

**Fig. 1:**
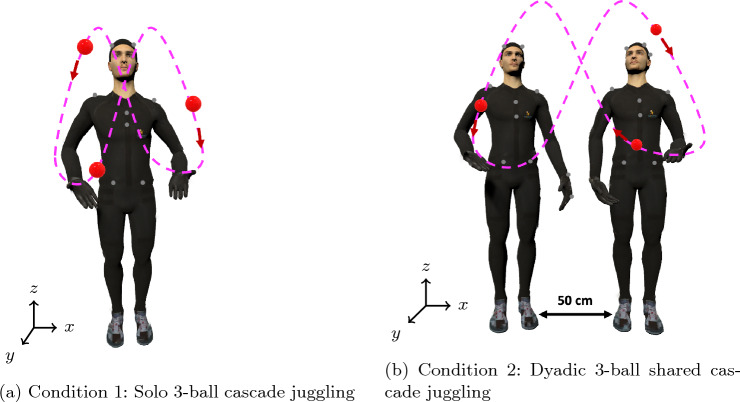
The juggling tasks involved in the two conditions of the experiment. Panel **a** shows Condition 1 (solo juggling), in which a single juggler performs a 3-ball cascade pattern. Panel **b** shows Condition 2 (dyadic juggling), in which two jugglers stand side by side approximately 50 cm apart and jointly perform a shared 3-ball cascade pattern, with the left juggler (partner) using their left hand and the right juggler (initiator) using their right hand. Dashed lines indicate the ball trajectories and filled circles represent the balls

The experiment had two conditions. In the first condition, the juggler had to perform a regular 3 ball cascade pattern (solo juggling, Fig. 1a), and in the second condition, the juggler was paired with another juggler with whom they performed a side-to-side shared 3 ball cascade pattern (dyadic juggling, Fig. 1b). The choice of participants in the dyad was made randomly. For the second pattern, both jugglers stood next to each other facing forward, and the juggler (partner) standing to the left of the other juggler used their left hand, while the other juggler (initiator) used their right hand. After each trial, the jugglers switched places, i.e., the initiator of the previous trial became the partner in the next trial, and vice-versa. In both the conditions, each trial started with the first throw from the right hand (specifically, in the dyadic juggling case, by the initiator). The participants did not observe one another’s performance in condition 1. The conditions were also undertaken in a counterbalanced order.

No instructions regarding juggling speed and height were given, and the participants were instructed to start juggling after they heard the phrase “GO!” by the experimenter. In each condition, the participant had to complete 10 trials. Each trial lasted until the experimenter gave a “STOP!” command at around 20 s or until the juggling pattern collapsed, whichever happened first. Every trial started with the jugglers standing at the same spot and, in condition 2, they stood $\approx 50\,\text {cm}$ apart (as illustrated in Fig. 1b).

### Data acquisition

Movements of the jugglers and the ball trajectories were recorded using a passive marker-based optoelectrical camera system (Vicon Motion Systems, Oxford, UK). 36 cameras (28 Vicon Vantage V5, and 8 Vicon Vero v1.3) were used and the data was collected at 240 Hz. The system was calibrated according to the manufacturer’s guidelines prior to data collection. Participants were taped with Pearl hard-base markers according to the upper body Plug-in-Gait marker set. Henrys smooth beanbag-style balls with ball diameters as 67 mm and weight 125 g were used as juggling balls. The three balls were covered with retro-reflective tape and defined as markers, to record the movements of the balls. The 3D trajectories of the passive markers were reconstructed using Nexus software. The coordinate system used was defined as follows: $\vec {x}-axis$ pointing left-to-right, $\vec {y}-axis$ along the antero-posterior axis, and $\vec {z}-axis$ along the vertical axis pointing upwards.

### Data processing and analysis

**Fig. 2:**
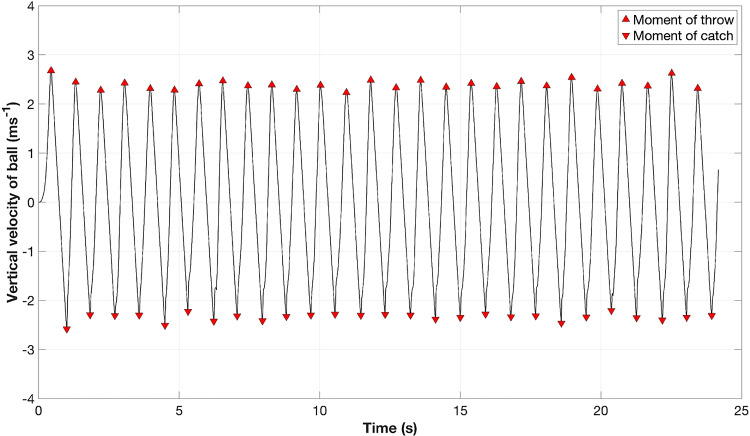
Vertical velocity profile of an example ball trajectory. The positive velocity peak corresponds to the ball release (throw), while the negative velocity peak corresponds to the ball interception (catch). The interval between two consecutive throw and catch markers represents the flight phase of the ball

**Fig. 3:**
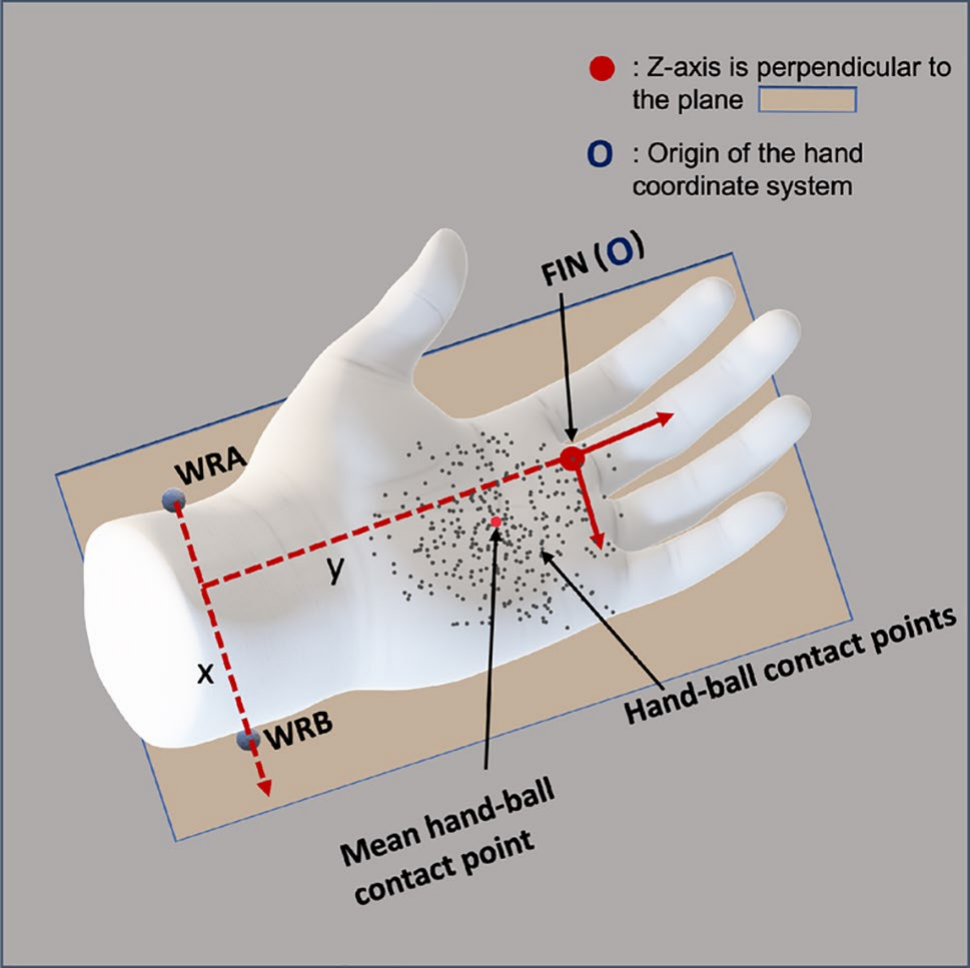
The hand representation in the analysis with $\vec {x}-axis$ pointing left-to-right, the $\vec {y}-axis$ pointing back-to-front, and the $\vec {z}-axis$ pointing up when the palm is facing upwards. The origin of this representation is at the FIN marker. The hand model shown is for illustrative purposes only and is not based on actual participant hand dimensions

Data processing and analyses were conducted using MATLAB R2024b (The MathWorks, Inc). The data was first filtered with a first-order two-way (zero-lag) Butterworth low-pass filter with a 22 Hz cutoff frequency to smoothen out the raw motion capture data and remove the high frequency jitters. Next, the individual ball trajectories during flight time (i.e., the time between a ball’s release from one hand and its subsequent catch by the other) were segmented by looking at the vertical velocity profile of the balls to identify the moments of throw and catch. A positive velocity peak denotes the moment of throw, and a negative velocity peak denotes the moment of catch, as illustrated in Fig. 2. In the same duration, the trajectory of the markers placed on the dorsal head of the 3${^{rd}}$ metacarpal (FIN), and on the two sides of the wrist (WRA, WRB: thumb-side and “little finger"-side) were also segmented. In order to have a better representation of the hand position, which considers the offset between the hand and ball markers at contact, we did the following corrections. First, we transformed the ball-hand contact points to a hand coordinate system, taking the $\vec {x}-axis$ pointing left-to-right, the $\vec {y}-axis$ pointing back-to-front, and the $\vec {z}-axis$ pointing upwards when the palm is facing upwards. The FIN marker was used as origin of the hand coordinate system (Fig. 3). This transformation of a global to a hand coordinate system of the hand-ball contact points enables detecting ball position relative to the hand. We then took the mean ball-contact position in this hand coordinate system separately for each hand and used this information to correct for the systematic offsets in ball-hand contact.

**Fig. 4:**
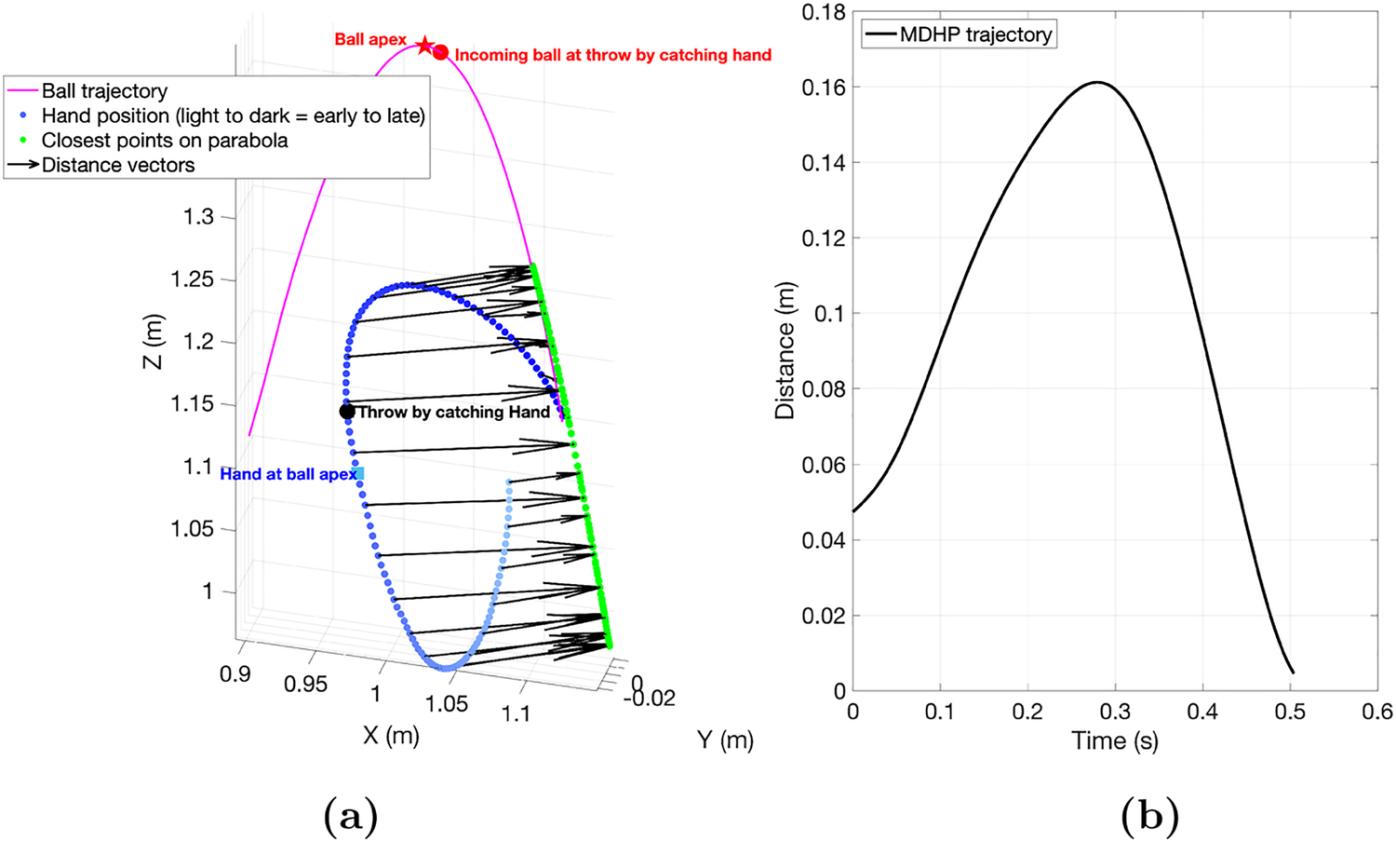
The figure in panel **a** demonstrates the evolution of hand and ball trajectory, for an example trajectory, in the duration between a ball’s release from one hand and its subsequent catch by the other. The arrows mark the minimum distance vectors from hand to ball’s future trajectory, plotted every 5 time samples apart (to avoid visualization clutter). The figure in panel **b** demonstrates the corresponding Minimum Distance of the Hand to the Parabola (MDHP) trajectory for the same example trajectory

This study explores to which extent anticipation of a ball’s trajectory based on internally available information during the throw affects the following catch of that particular ball. Following the method proposed in Slupinski et al. (2018), we examined how predictions based on information from the throwing hand affects the timing of the catching hand’s movements. By leveraging the empirical principle that goal-directed trajectories exhibit minimal jerk (Flash and Hogan 1985; Hogan 1984; Hoff 1992; Fligge et al. 2012; Desmurget et al. 1998), we investigated how early jugglers achieve such smooth, goal-directed hand movements in solo juggling (condition 1) compared to when they lack throwing information from the opposite hand (condition 2). This approach evaluates the shortest distance between the hand and the ball’s future trajectory over time (Fig. 4), with Figure S1 (Online Resource 1) comparing the average MDHP profiles across conditions. Using this one-dimensional trajectory data (MDHP or Minimum Distance of the Hand to the Parabola), the smooth (goal-directed) phase is determined by fitting it to the minimum jerk equation, where jerk is defined as the third derivative of position with respect to time. A minimum jerk trajectory, $J_{min}$, minimizes the sum of the squared jerk values along the object’s path between an initial time $t_o$ and a final time $t_f$:

$$ J_{\text {min}} = \min _{x(t)} \int _{t_0}^{t_f} \left( \frac{d^3 x(t)}{dt^3} \right) ^2 dt $$

**Fig. 5:**
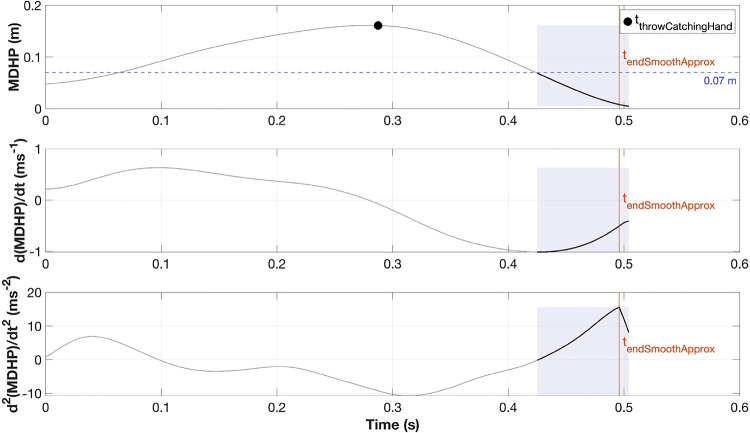
Identification of $t_{endSmoothApprox}$ in the MDHP trajectory. MDHP versus time with shaded region where MDHP < 0.07 m, threshold line at 0.07 m (blue dashed), and marker indicating the throw by the catching hand (black filled circle). The vertical line marks $t_{endSmoothApprox}$, defined as the first peak deceleration in the MDHP trajectory, obtained by looking at the second derivative of the MDHP trajectory and identifying the first peak within the shaded region

To prepare for analysis, we first filtered the MDHP trajectory with a third-order (zero-lag) Butterworth low-pass filter with a 12 Hz cutoff frequency. We next segment the MDHP trajectory into its constituent functional phases for analysis. The first phase in the segmentation constitutes the approximation to the parabola phase and the next phase is the interception along the parabola phase. The first approximation phase starts from the time when the ball leaves the other hand and ends at the end of the goal-directed movement phase. The next phase marks the start of the interception phase, where the hand could move along the trajectory of the ball and make the catch. This time point $t_{endSmoothApprox}$ is the time when the hand is still approaching the ball’s parabola but very slowly, and is calculated as follows (as illustrated in Fig. 5):

- **Definition of proximity criterion**: The hand is considered to follow the ball trajectory closely enough for a successful catch when the MDHP fell below 0.07 m.
- **Temporal search window**: To ensure that $t_{endSmoothApprox}$ is identified only after the previous ball has been released, the start of the search window is defined as the later of: a) the moment of throw of the ball already in hand, or b) the first time point at which MDHP dropped below 0.07 m.
- **Operational definition of** $t_{endSmoothApprox}$: Within this window, the first peak deceleration in the MDHP trajectory is identified and taken as $t_{endSmoothApprox}$, marking the onset of the interception phase.

### Dependent variables

**Fig. 6:**
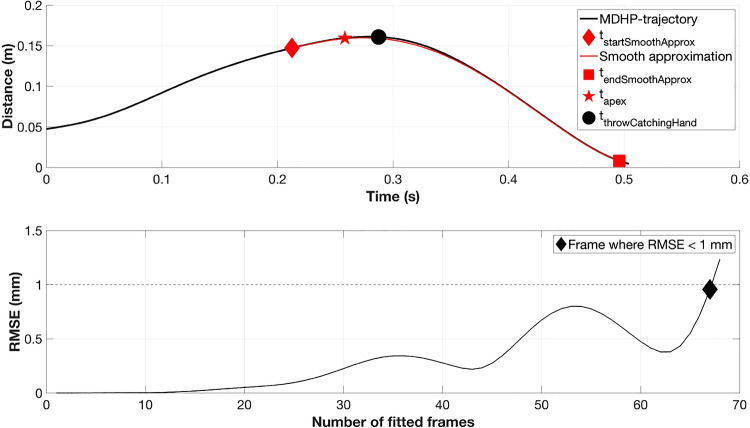
Example of a minimum jerk fit on the MDHP trajectory for a solo condition. Minimum jerk trajectories are plotted for each time step before $t_{endSmoothApprox}$ and continue until the RMSE of the fit exceeds 1 mm, which marks the end of the fit. The resulting minimum jerk trajectory is the smooth approximation curve (as illustrated), which functionally starts from $t_{startSmoothApprox}$ and ends at $t_{endSmoothApprox}$. In addition, $t_{apex}$ and $t_{throwCatchingHand}$ denote the moment when the incoming ball reaches its apex and the moment when the catching hand is emptied before intercepting the incoming ball, respectively

The dependent variable, the start of the smooth approximation phase, was determined following the method described in Slupinski et al. (2018). The variable, ($t_{startSmoothApprox}$), reflects the earliest time at which the catcher’s prediction of the ball’s trajectory has become sufficiently accurate to guide a smooth, goal-directed movement – that is, the time from which corrective adjustments to the approach trajectory are no longer observed. It is the earliest time at which the MDHP-trajectory satisfied the minimum jerk criterion (Flash and Hogan 1985; Hogan 1984). The minimum jerk trajectories for each time step before $t_{endSmoothApprox}$ were calculated until the root mean square error (RMSE) in position exceeded 1 mm, considering the spatial resolution of a general motion capture system (Slupinski et al. 2018) (as illustrated in Fig. 6). Figure S2 (Online Resource 1) presents an additional sensitivity analysis of the choice of the RMSE threshold.

To meaningfully eliminate variability by enabling comparison in flight times and, subsequently, the relevant temporal variables in our analysis, we have normalized time to the ball’s apex and the throw of ball by the catching hand, respectively. The apex was chosen as the anchor point under the assumption that jugglers use the zenith of the ball’s flight as a key reference for their prediction, since this is where the ball’s vertical velocity is zero and trajectory information is clustered there (Van Santvoord and Beek 1994; Amazeen et al. 1999; Huys and Beek 2002; Dessing et al. 2012; Beek and Lewbel 1995). Additionally, absolute time, governed by the external clock, reflects the real world temporal evolution of each event. However, the motor control processes underlying skilled actions such as juggling are often organized relative to internal timing mechanisms that operate independently of physical time. By normalizing each throw’s time axis, we map the movement data onto a common internal timescale, capturing the phase progression of each action. This also allows us to compare the onset of the smooth approximation phase with different events within the trajectory, ensuring that the event in question is temporally aligned at the same point for both conditions. In this way, we can examine whether the hand’s approach to the ball is consistently structured with respect to the internal dynamics of an event in the juggling cycle, rather than tied to specific points on the external timeline.

All parameters refer to time (in s) within a single trajectory, with the flight duration defined as the time between $t_{throw}$ to $t_{interception}$, and each measure is expressed as a percentage of the remaining flight duration from the reference point to the interception.

The timing of the outgoing throw relative to the apex of the incoming ball’s flight was defined as:

$$ t_{\text {throwNormApex}} = \frac{ t_{\text {throwCatchingHand}} - t_{\text {apex}} }{ t_{\text {interception}} - t_{\text {apex}} } \times 100\,\% $$

The onset of the smooth approximation phase ($t_{\text {startSmoothApprox}}$) relative to the apex was defined as:

$$ t_{\text {startSmoothNormApex}} = \frac{ t_{\text {startSmoothApprox}} - t_{\text {apex}} }{ t_{\text {interception}} - t_{\text {apex}} } \times 100\,\% $$

The timing of the apex relative to the outgoing throw that frees the catching hand was computed as:

$$ t_{\text {apexNormThrow}} = \frac{ t_{\text {apex}} - t_{\text {throwCatchingHand}} }{ t_{\text {interception}} - t_{\text {throwCatchingHand}} } \times 100\,\% $$

Finally, the onset of the smooth approximation phase relative to the outgoing throw was calculated as:

$$ t_{\text {startSmoothNormThrow}} = \frac{ t_{\text {startSmoothApprox}} - t_{\text {throwCatchingHand}} }{ t_{\text {interception}} - t_{\text {throwCatchingHand}} } \times 100\,\% $$

### Task-level spatial covariates

Four spatial variables were computed from the segmented ball trajectories described in Sect. “Data processing and analysis”, with the catching hand position extracted over the corresponding flight interval. Toss height (*tossHeight*) was defined as the difference in vertical position between the ball’s apex and its release point. Trajectory width (*trajWidth*) was defined as the absolute lateral displacement of the ball between release and interception — this captures the spatial extent of the ball’s path across conditions. Lateral excursion (*lateralExcursion*) was defined as the total range of the catching hand’s lateral position ($\vec {x}-axis$) over the flight interval, capturing the sideward component of the hand’s movement. Vertical excursion (*verticalExcursion*) was defined as the total range of the catching hand’s vertical position ($\vec {z}-axis$) over the same interval, capturing the hand’s vertical movement extent. All four variables were added as fixed-effect covariates in a linear mixed-effects model (see Sect. “Ruling out confounds: linear mixed-effects model”).

### Statistical analysis

JASP version 0.19.3 (Jasp Team 2025) was used for all statistical analyses. For all tests, the significance level was set to $\alpha = 0.05$. Due to skewness in the individual participant data, the median for all parameters were calculated for each participant. These participant-level median values served as the dependent variables for all inferential analyses. The necessary normality assumption to run a paired *t*-test was checked using the Shapiro-Wilk test, which indicated no significant deviations from normality. Finally, a one-tailed paired *t*-test was used to compare the dependent variables between two conditions. Statistical effect sizes were determined using Cohen’s *d* measure and they were corrected for correlation between observations (Lakens 2013).

A priori power analysis (G*Power) was conducted with a conservative effect size of 0.8 (following Slupinski et al. (2018) but allowing extra variability due to the juggling task), $\alpha = 0.05$, and power = 0.95, yielding a required sample size of 18 participants.

## Results

### Start of smooth approximation

**Fig. 7:**
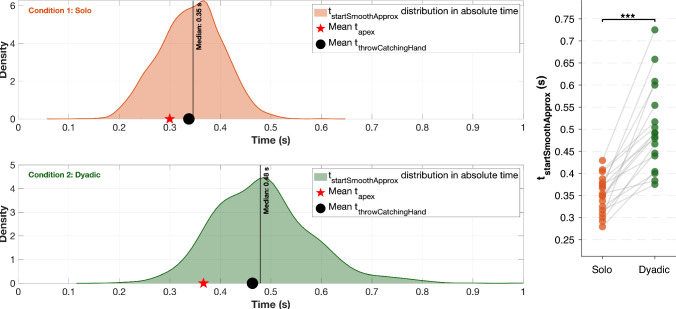
Distribution of $t_{startSmoothApprox}$ for condition 1 & 2 in non-normalized time. Red markers indicate the mean ball apex times, and black markers indicate the mean times of the ball throws preceding interception by the intercepting hand. The vertical line denotes the median onset timing. The right panel shows individual participant median values for each condition, with lines connecting each participant’s paired observations

Figure 7 shows the distribution of onset timing of smooth approximation phase ($$t_{startSmoothApprox}$$) in absolute time. It is evident from the figure that, in condition 1 (*mean *±* SE of participant-wise medians*: $$0.352 \pm 0.010$$ *s*), $$t_{startSmoothApprox}$$ started significantly earlier compared to condition 2 (*mean* ± *SE of participant-wise medians*: $$0.502 \pm 0.023$$ *s*)[$$t_{17}$$ $$ = -7.502$$, *p*$$ < 0.001$$, Cohen’s *d* =$$ -1.809$$]. Interestingly, the onset of $$t_{startSmoothApprox}$$ occurred more frequently before the incoming ball’s apex in condition 1 than in condition 2.

**Fig. 8:**
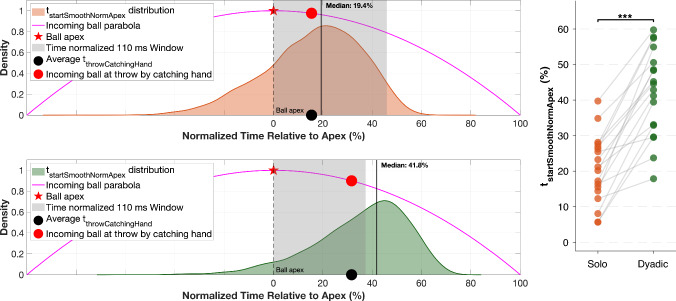
Distribution of normalized onset timing of smooth approximation phase relative to the incoming ball’s apex, $t_{startSmoothNormApex}$, for condition 1 and 2. 0 % indicates the event of the ball reaching its apex and 100% marks the interception. The shaded area represents a 110 ms visuomotor response window, adjusted to the average flight duration for each condition. Condition 1 shows an earlier onset of the smooth approximation phase relative to the apex compared to condition 2. The incoming ball parabola is plotted for illustration purposes, indicating different events in the trajectory, i.e., the ball apex and the incoming ball’s position at the throw by the catching hand. The vertical line denotes the median onset timing. The right panel shows individual participant median values for each condition, with lines connecting each participant’s paired observations

Since ball flight times varied across trials, aligning movements to a common internal timescale allows direct comparison of event timing across conditions. In Fig. 8, we visualize the normalized timing of smooth approximation onset ($$t_{startSmoothNormApex}$$) with respect to the incoming ball’s apex and expectedly observe that the start of the smooth approximation phase with respect to apex was significantly earlier [$$t_{17}$$ $$ = -9.033$$, *p*$$ < 0.001$$, Cohen’s *d* =$$ -1.929$$] in the solo juggling scenario (*mean *±* SE of participant-wise medians*: $$20.489 \pm 2.246 $$) compared to its dyadic counterpart (*mean *± *SE of participant-wise medians*: $$42.073 \pm 2.880 $$). Here, 0% corresponds to the moment when the incoming ball reaches its apex, and 100% marks the actual interception. To account for the time required to initiate corrective movements, we included an additional window of 110 ms, based on established findings in interception research (Brenner et al. 1998; Soechting and Lacquaniti 1983; Brenner and Smeets 1997; Fooken et al. 2024), which show that when a moving target is visually perturbed, the hand’s corrective response latency is approximately 110 ms. In Fig. 8, this analysis window is time-normalized relative to the average post-apex trajectory duration for each condition as illustrated below:

$$\begin{aligned}&\text {Normalized 110 ms window} \nonumber \\&\qquad = \frac{ 0.110 }{ \text {mean post-} t_{\textrm{apex}} \text { trajectory duration}} \times 100\% \end{aligned}$$

This normalization is motivated by visuomotor delays: if jugglers were merely reacting to the ball apex and updating their movement plan based on visual information at that moment, any resulting corrective movement would be expected to occur no earlier than approximately 110 ms later.

In contrast, it can be observed that the onsets of the smooth-approximation phase fall mostly into this window in the solo condition compared to the dyadic condition.

### Testing competing hypotheses

**Fig. 9:**
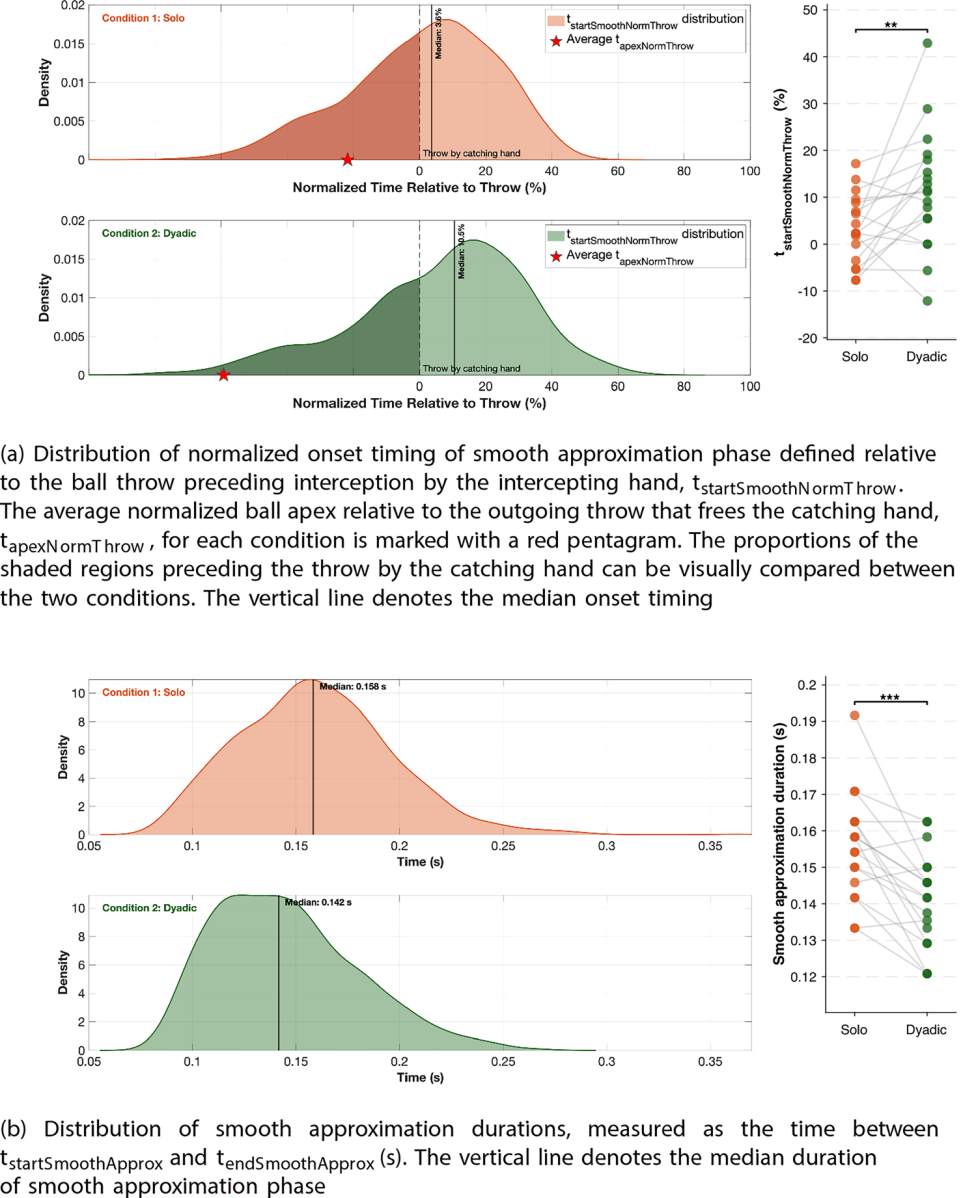
Comparison of onset timing normalized to the outgoing throw by the catching hand, and duration of the smooth approximation phase. The right panels for both **a** and **b** show individual participant median values for each condition, with lines connecting each participant’s paired observations

In both conditions, jugglers make an outgoing throw to empty the catching hand for the next catch. If the preparation time for the catching action were always the same after freeing the hand, no difference would be expected between conditions. However, Fig. 9a illustrates that the timing of the onset of the smooth approximation phase ($$t_{startSmoothNormThrow}$$) cannot be explained simply by when the catching hand is freed. The onset of the smooth phase relative to the outgoing throw is significantly later [$$t_{17}$$ $$ = -2.672$$, *p*$$ = 0.008$$, Cohen’s *d* = $$ -0.779$$] in the dyadic condition (*mean *± *SE of participant-wise medians*: $$11.454 \pm 2.992$$) compared to the solo condition (*mean *±* SE of participant-wise medians*: $$3.215 \pm 1.734$$).

The duration of the smooth approximation phase, defined as the interval from $$t_{startSmoothApprox}$$ to $$t_{endSmoothApprox}$$, was also analyzed to test whether longer absolute flight times in the dyadic condition could account for the delayed onset observed previously. Figure 9b shows that the duration of the smooth phase was significantly shorter [$$t_{17}$$ $$ = 4.570$$, *p*$$ < 0.001$$, Cohen’s *d* =1.076] in the dyadic condition (*mean ± SE of participant-wise medians*: $$0.141 \pm 0.003$$ *s*) compared to the solo condition (*mean ± SE of participant-wise medians*: $$0.156 \pm 0.003$$ *s*).

### Ruling out confounds: linear mixed-effects model

One could argue that the transition from the solo to the dyadic condition leads to a change in the spatiotemporal organisation of the task (despite the pattern looking the same structurally). Therefore, the differences in the timing onset of predictive control could just be a by-product of the trajectory differences. Since juggling expertise can be considered a spectrum, one could also argue that, in the dyadic condition, skill asymmetries between the pair or physical differences could explain the differences in the dependent variables between the two conditions. To ensure our analysis was statistically robust to these potential confounders, we conducted linear mixed-effects modeling in which *tossHeight*, *trajWidth*, *lateralExcursion*, and *verticalExcursion* were included as fixed-effect covariates alongside *Condition*, while accounting for the hierarchical structure of the data using random intercepts and random slopes for Condition across participants (*ParticipantID*), and random intercepts for dyads (*PairID*).

The model took the following form:

$$\begin{aligned} \begin{aligned} \textit{Dependent Variable} \sim \;&\textit{Condition} + \textit{trajWidth} + \textit{tossHeight} \\&+ \textit{lateralExcursion} + \textit{verticalExcursion} \\&+ (1+\textit{Condition} \mid \textit{ParticipantID}) \\&+ (1 \mid \textit{PairID}) \end{aligned} \end{aligned}$$

The models for $t_{startSmoothApprox}$ and $t_{startSmoothNormThrow}$, however, produced singular fits, indicating overparameterisation given the available sample of 18 participants (Barr et al. 2013); random intercepts only were therefore retained for these two outcomes. For $t_{startSmoothNormApex}$ and duration of smooth approximation, the random slope models converged. The results of this analysis (see Online Resource 1, Table S1) corroborated the significant influence of *Condition*: across all dependent measures, the effect of *Condition* remained statistically significant after adjustment for all four covariates, demonstrating that the observed differences between solo and dyadic juggling are not a by-product of task-related geometry nor of skill or physical asymmetries between dyad partners. Although geometric factors systematically influenced timing, they did not account for the condition effects.

### General kinematic differences between condition 1 and condition 2

In our study, Condition 2 differed from Condition 1 in several task level kinematic characteristics. Dyadic juggling is characterized by higher toss heights [$$t_{17}$$ $$ = -6.376$$, *p*$$ < 0.001$$, Cohen’s *d* =$$ -1.523$$] and wider ball trajectories [$$t_{17}$$ $$ = -9.753$$, *p*$$ < 0.001$$, Cohen’s *d* =$$ -3.913$$] compared to its solo counterpart. These changes were accompanied by longer ball flight times and a slower overall juggling rhythm. In addition, to measure juggling consistency, dwell ratio has been defined as the proportion of the juggling cycle during which a hand remains in contact with the ball between successive catches or throws (Beek and Turvey 1992). We observe that the dwell ratio was significantly higher [$$t_{17}$$ $$ = -6.411$$, *p*$$ < 0.001$$, Cohen’s *d* =$$ -1.511$$] in the dyadic condition (group level mean combined dwell ratio, $$\bar{r} = 0.779 \pm 0.047$$, $$mean \pm SD$$) than in the solo condition (group level mean dwell ratio, $$\bar{r} = 0.701 \pm 0.058$$, $$mean \pm SD$$), indicating that a larger proportion of the juggling cycle was spent with the ball in the hand.

## Discussion

In this study, we wanted to investigate the relevance of internal and tactile information from one’s own throwing movement for the planning of anticipatory movement parts of a subsequent catch. To this end, we employed a paradigm that included both self-generated actions (solo juggling) and externally generated actions (dyadic juggling). The dyadic condition serves as our best approximation of a situation in which internal information about the throw, such as proprioceptive, tactile, and efferent cues, is unavailable, while keeping the juggling pattern structurally similar to the solo condition. Our specific aim was to investigate whether the absence of internal information about the throw, such as when the throw is made by a partner, affects the timing of hand movement planning for the subsequent catch. The answer to this question is not particularly straightforward in this context, since planning runs parallel with ongoing movement control. The juggler must release the ball currently held in the hand before initiating the catch movement, all while maintaining the temporal stability of the juggling rhythm. Our underlying assumption was that anticipatory behavior would be reflected in the timing at which the hand smoothly moves toward the trajectory of the incoming ball. Our findings align with prior work showing earlier planning conclusion and movement initiation for self-generated discrete throws (Hagenfeld et al. 2022; Slupinski et al. 2018), but critically, we demonstrate this effect in a continuous coordinated task. This suggests that predictive control strategies are not limited to discrete reaches, but are continuously adapted based on the internal vs. external origin of motor timing information, even in non-discrete, continuous tasks like toss juggling.

Unlike Slupinski et al. (2018) and Hagenfeld et al. (2022), we could not fully control for ball trajectory flight time and flight distance due to the dynamic and rhythmic nature of the juggling task. However, we accounted for this variability in our analysis by normalizing times to flight duration. One could argue that this variation in flight time provided an opportunity to test whether having more time available leads to earlier movement onset. Since the hand is emptied in both conditions, following the alternative explanation, the preparation time should be similar if freeing the hand was the only constraint. Following this explanation, we should not see a significant shift in the onset of smooth phase distributions between the two conditions. Figure 9a shows otherwise and rules out the alternative explanation that the timing difference in $t_{startSmoothApprox}$ is solely due to when the hand is freed for the catch. The difference in timing onset between the two conditions is likely due to the absence of internal motor information. When jugglers throw the ball themselves, as in the solo condition, they have access to efferent and proprioceptive signals that help them plan the catch. In the dyadic condition, this information is missing, which likely shifts the initiation of predictive control. Additionally, if the timing difference was only a result of having more time as in condition 2, then the duration of the smooth, goal-directed phase should be similar or longer. However, Fig. 9b rejects the alternative explanation that the delayed onset of the smooth phase in dyadic juggling is solely due to reduced time pressure from longer ball flights. The shorter smooth phase indicates that jugglers delayed committing to predictive control even when more time was available. This supports the interpretation that the absence of efferent and proprioceptive information from self-generated throws is associated with delayed initiation of prediction in the dyadic condition. Interestingly, this reflects broader findings that relying only on external sensory motion cues can limit predictive accuracy. For instance, Kreyenmeier et al. (2022) showed that when people had to predict the reappearance of an accelerating target using visual information alone, they systematically mistimed their interception once the target was briefly occluded. Taken together, our results indicate that it is not simply the amount of time available that limits movement planning, but rather the level of predictive certainty.

It may seem surprising that adjustments to movement can occur right at the start, given the physical distance between the brain and the arm muscles, as well as the inherent physiological delay in muscle activation. Although responses to visual disturbances can be initiated in as little as $\sim$110 ms (Brenner et al. 1998; Brenner and Smeets 1997), this is still relatively slow compared to the total duration of a catching movement. In fact, even movements traditionally considered ballistic are under continuous visual control. Additionally, the acceleration of the hand also modulates in real-time in response to target speed changes, with effects visible after roughly 200 ms (Brenner et al. 1998). The apex of the ball’s trajectory was chosen as an anchor point for analyzing the onset of predictive control because it provides an ideal moment for jugglers to update their internal estimates about the ball’s flight. At the apex, the ball’s vertical velocity is momentarily zero, making its position and path maximally stable and predictable. Prior studies have shown that jugglers naturally rely on visual information near the apex; novices often directly fixate on each ball’s apex to judge its landing point, while experienced jugglers implicitly use apex cues to maintain timing and consistency (Van Santvoord and Beek 1994; Huys and Beek 2002; Dessing et al. 2012; Amazeen et al. 1999). Even when vision is restricted to a narrow region near the apex, skilled jugglers can maintain the pattern, demonstrating how the apex contains key predictive information about trajectory and timing. This is directly relevant to how we interpret the 110 ms window shown in Fig. 8. If jugglers were relying only on this apex information to adjust their movement, any correction triggered around this point would require at least about 110 ms to appear in the hand’s motion, given typical visual processing and neuromuscular delays (Brenner et al. 1998; Brenner and Smeets 1997). In juggling, this dynamic plays out clearly as the apex may act as a reliable visual reference for feedforward prediction, but jugglers can also make fine-tuned corrections in the final phase before interception. The 110 ms window reflects this limit; any visual corrections initiated after this point are unlikely to influence the outcome in time. Thus, when the onset of the smooth approximation phase $t_{startSmoothApprox}$ falls predominantly within this window, as seen in the solo condition, this is consistent with jugglers relying more on preplanned, internally generated predictions. In contrast, when $t_{startSmoothApprox}$ shifts later, as in the dyadic condition, this pattern is consistent with increased dependence on continuous visual feedback to refine the interception trajectory. This distinction aligns with the broader view that skilled juggling combines both feedforward and feedback control, supported by efference copies of outgoing motor commands and internal forward models (Miall and Wolpert 1996) that help predict the consequences of the movement in real time.

The observed increase in dwell ratio in the dyadic condition further supports this interpretation at the level of overall task organization. After a ball has been caught, the hand must move from the catch position to the subsequent throw position and, following the throw, travel back toward the next catch location. From a purely geometric perspective, this symmetry might suggest a dwell ratio of approximately 0.5. However, several factors make a longer dwell time advantageous. Increasing the dwell ratio provides additional time to stabilize and control the ball in the hand, which can improve throw accuracy. In addition, based on Claude Shannon’s juggling theorem (Shannon 1993; Beek and Lewbel 1995), a higher dwell ratio consequently reduces $\omega$ (Kalvan and Lewbel 2018), defined as the average number of balls simultaneously in flight per arc. Reducing $\omega$ lowers the likelihood of spatial and temporal overlap between balls and thereby reduces the risk of collisions. In the context of dyadic juggling, where internal information about the throw is unavailable, an increased dwell ratio may function as a temporal buffer, allowing additional time for sensory-based adjustments before initiating the next throw. Thus, the higher dwell ratio observed in dyadic juggling reflects not only altered kinematics but may also indicate an adaptive reorganization of timing under reduced predictive certainty.

While our study was designed to investigate the role of internal information in anticipatory motor planning, there are limitations to the interpretations that can be drawn. In the dyadic condition, participants lacked access to proprioceptive, tactile, and efferent signals associated with self-generated throws, which we refer to collectively as internal information. However, we did not isolate the individual contributions of these signals, and their respective roles in predictive planning in the context of toss juggling remain an open question. Additionally, we acknowledge that dyadic juggling introduces social and cognitive demands beyond the absence of internal motor cues. Although our analysis included pair group as a random effect, demonstrating that the main effect of condition remained significant, we cannot fully disentangle these broader influences within the current paradigm. Nonetheless, the structural similarity between conditions, combined with consistent task demands and participant pairing, likely support our interpretation that the absence of integrated internal information contributed to delayed predictive control in the dyadic condition.

Finally, to summarize, the goal of this study was to demonstrate that the internal information from the juggler’s throwing hand plays an important role in determining the timing of the catching hand’s movement. In a juggling task, where the ball can be caught at any point along its parabolic trajectory, accurate and early prediction of the ball’s path is essential for maintaining stability in the juggling pattern. We argue that jugglers use information from their own throw (when available) to make those predictions early, enabling a timely and smooth interception. Our findings highlight the relationship between sensory input and motor planning, emphasizing the importance of efferent, proprioceptive, and tactile information in enabling smoother and more efficient hand movements in complex sensorimotor tasks like juggling. The juggling example may further illustrate how the brain brings together feedforward and feedback control. Feedforward predictions based on efference copy and internal forward model allow the catching movement to be initiated early and in the right general direction, while feedback (mainly visual, especially near the apex or during ball flight) provides ongoing corrections to fine tune the movement, which is under control of the perceived position of the target. When internal information are available (as in solo juggling), the system generally relies more on predictive control; when they are absent (as in catching someone else’s throw in dyadic juggling), the system increases reliance on prospective control. Ultimately, jugglers achieve a seamless integration of these processes. They demonstrate that even a task often perceived as rapid and rhythmic still involves guidance by sensory input, be it the “internal” vision of one’s own throw or the “external” sight of the ball in flight. This synergy between sensory input and motor planning is fundamental not only to juggling, but to a wide range of interceptive and interactive motor behaviors in daily life.

## Supplementary Information

Below is the link to the electronic supplementary material.

Supplementary file 1 (pdf 672 KB)

## Data Availability

Data used in this study will be made public at osf.io/u8b3r.
