# Supplementary material for: The earlier you know, the smoother you act: anticipatory control in solo and dyadic juggling
Source: Exp Brain Res. 2026 May 12;244(6):117. doi: 10.1007/s00221-026-07311-z (PMC13167880; doi:10.1007/s00221-026-07311-z)
Supplement: Supplementary file 1 — (pdf 672 KB) [file 221_2026_7311_MOESM1_ESM.pdf]

Supplementary information for

The Earlier You Know, the Smoother You Act: Anticipatory Control in Solo and Dyadic Juggling

Abir Chowdhury*

Heiko Maurer

Alap Kshirsagar

Kai Ploeger

Jan Peters

Hermann Müller

Lisa Katharina Maurer

*Correspondence concerning this article should be addressed to Abir Chowdhury, Neuromotor Behavior Laboratory, Department of Psychology and Sport Science, Justus Liebig University, Giessen, Germany. Email: abir.chowdhury@sport.uni-giessen.de

Journal: Experimental Brain Research

Table S1

Covariate-Adjusted Linear Mixed-Effects Model for Smooth Approximation Timing

Models were fitted in R (version 4.5.3) using the *lme4* (Bates et al., 2015) and *lmerTest* (Kuznetsova et al., 2017) packages. All models were fitted by restricted maximum likelihood (REML) with Kenward-Roger approximation (Kenward & Roger, 1997) for estimating denominator degrees of freedom and standard errors for fixed-effects, which provides bias-corrected inference for models with small number of clusters. Fixed effects included *Condition* (solo vs dyadic), trajectory width (*trajWidth*), toss height (*tossHeight*), and lateral (*lateralExcursion*) and vertical excursion (*verticalExcursion*) of the catching hand. Random intercepts were included for participant (*ParticipantID*) and dyad (*PairID*) in all models. For *tStartSmoothNormApex* and Duration of smooth approximation (*smoothDurationAbs)*, a random slope for *Condition* by participant was additionally included as these models converged without singularity; random slope models for *tStartSmoothApprox* and *tStartSmoothNormThrow* produced singular fits and were therefore not retained (Barr et al., 2013). Condition effects remained significant across all four outcomes after covariate adjustment. VIF values ranged from 1.08 to 1.40 across all models, indicating negligible multicollinearity. Multicollinearity among fixed-effect predictors was assessed using variance inflation factors (VIF) computed via the vif() function from the *car* package (Fox & Weisberg, 2019) in R.

Predictor	tStartSmoothApprox	tStartSmoothNormApex	tStartSmoothNormThrow	smoothDurationAbs
	β (SE), t	β (SE), t	β (SE), t	β (SE), t
**Fixed effects**				
Intercept	0.239 (0.010), 22.79***	24.713 (3.876), 6.38***	−88.468 (5.273), −16.80***	0.120 (0.006), 18.69***
Condition (solo)	−0.038 (0.009), −4.31***	−8.075 (3.527), −2.29*	+38.189 (4.558), 8.38***	+0.023 (0.005), 4.47***
trajWidth	0.049 (0.006), 8.41***	26.234 (2.257), 11.67***	65.293 (2.825), 23.14***	0.014 (0.004), 3.12**
tossHeight	0.430 (0.008), 53.87***	26.469 (3.129), 8.54***	37.053 (3.914), 9.49***	0.000 (0.006), 0.01 ns
lateralExcursion	0.072 (0.009), 8.23***	27.702 (3.436), 8.09***	32.759 (4.305), 7.62***	−0.025 (0.007), −3.66***
verticalExcursion	−0.144 (0.010), −14.96***	−45.182 (3.761), −12.07***	42.204 (4.715), 8.96***	0.041 (0.008), 5.45***
**Random effects (SD)**				
ParticipantID intercept	0.020	5.420	10.031	0.009
ParticipantID slope (Condition)	_	1.899	_	0.006
PairID	0.021	8.112	10.617	0.011
Residual	0.043	16.586	20.791	0.033
**Model specifics**				
Random slope for Condition	No	Yes	No	Yes
VIF range	1.08 – 1.34	1.08-1.33	1.08-1.33	1.10-1.40
Kenward-Roger df (Condition)	~21	~15	~21	~16

Note: β = unstandardised fixed-effect coefficient; SE = standard error; t = Kenward-Roger t-statistic. Condition (solo) reflects the effect of solo relative to dyadic juggling and is highlighted in light gray. trajWidth = lateral ball displacement release to interception; tossHeight = vertical distance release to apex; lateralExcursion and verticalExcursion = range of catching hand movement along x- and z-axes. Random effects reported as standard deviations. "—" indicates random slope not included (singular fit). VIF range = minimum–maximum variance inflation factor across all predictors. Kenward-Roger df for Condition is lower for models with random slopes (tStartSmoothNormApex ~15, smoothDurationAbs ~16) reflecting the more conservative test when between-participant variability in the Condition effect is explicitly modelled.

Exact p-values for the Condition effect: tStartSmoothApprox $p < .001$, tStartSmoothNormApex $p = .037$, tStartSmoothNormThrow $p < .001$, smoothDurationAbs $p < .001$.

* $p < .05$, ** $p < .01$, *** $p < .001$, ns = not significant.

Figure S1

Average MDHP Trajectory: Solo vs Dyadic

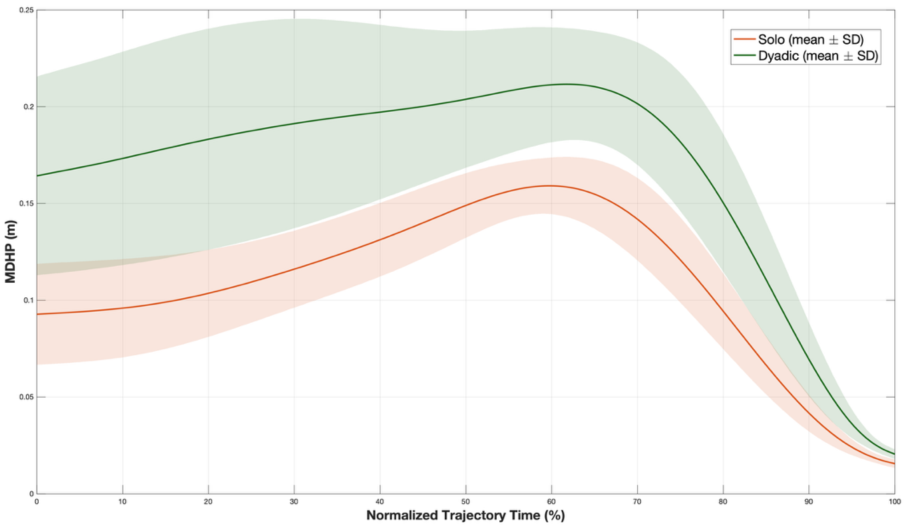

To allow comparison of MDHP trajectory shape across conditions, each MDHP trajectory was time-normalized to 0 - 100% of its total flight duration by linear interpolation onto a common grid (resolution set to the longest trajectory in the dataset in order to avoid having to downsample any trajectory and losing information). Trajectories were then averaged within each participant (solo) or pair (dyadic) across all trials to produce a participant-level or pair-level mean MDHP profile. Grand mean and between-participant/pair standard deviation were then computed across all 18 solo participants and 9 dyadic pairs separately, and plotted as a function of normalized trajectory time.

Figure S2

<u>Sensitivity Analysis: Effect of RMSE Threshold</u>

To assess robustness to the choice of RMSE threshold, we repeated the analysis across a 12-fold range of threshold values (0.25 – 3 mm). Across all tested values, the relationship between solo and dyadic juggling was preserved. The dotted vertical line in each figure marks the (conservative) 1 mm threshold used in the main analysis, which follows Slupinski et al. (2018) and reflects the general spatial resolution of a motion capture system.

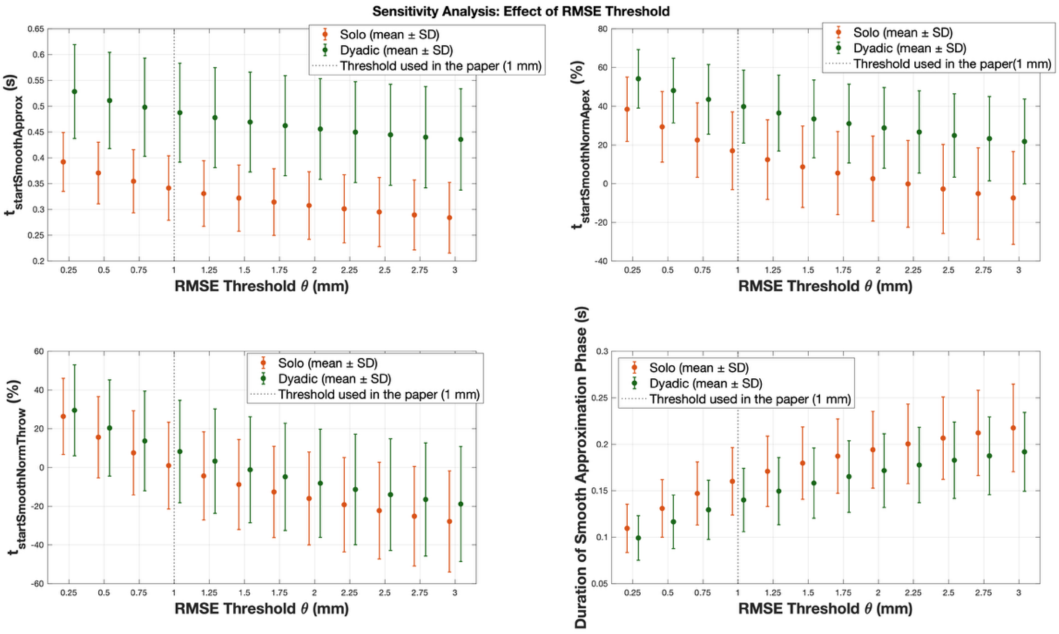

References:

Bates, D., Maechler, M., Bolker, B., & Walker, S. (2015). Fitting linear mixed-effects models using lme4. Journal of Statistical Software, 67(1), 1–48.

Barr, D. J., Levy, R., Scheepers, C., & Tily, H. J. (2013). Random effects structure for confirmatory hypothesis testing: Keep it maximal. Journal of Memory and Language, 68(3), 255–278.

Kuznetsova, A., Brockhoff, P. B., & Christensen, R. H. B. (2017). lmerTest package: Tests in linear mixed effects models. Journal of Statistical Software, 82(13), 1–26.

Fox, J., & Weisberg, S. (2019). An R companion to applied regression (3rd ed.). Sage.

Kenward, M. G., & Roger, J. H. (1997). Small sample inference for fixed effects from restricted maximum likelihood. Biometrics, 53(3), 983–997.

Slupinski, L., de Lussanet, M. H., & Wagner, H. (2018). Analyzing the kinematics of hand movements in catching tasks—An online correction analysis of movement toward the target's trajectory. Behavior Research Methods, 50(6), 2316-2324.
